# The Therapeutics of Sexual Weakness

**Published:** 1894-09

**Authors:** 


THE THERAPEUTICS OF SEXUAL WEAKNESS.—The query propounded by a member of the drug trade, in the May number of the *Summary*, who is not seriously humiliated by the condition resulting from his depravity, will hardly be entirely and satisfactorily cleared away, even though the remarks of the editor are very *apropos* and eminently in the right line. The fact is, that very same query is asked of almost every doctor in the land, and few there are who offer the explanation, counsel, and substantial aid which satisfy the patient. Take the case of the drugman who wants to know what he can do to restore his sexual apparatus to a condition compatible with health, and which will insure a state that is not on the verge of bankruptcy at all times. Now this case has this one symptom that is ever present and occasions much mental inquietude, that of seminal emissions. This sensible evidence of inability of the body to prevent this loss is all powerful in alarming the victim that a serious drain is being made on the body. To rightly judge of this physiological act, it is necessary to note the anatomy and normal action of the parts concerned.

In most animals seminal emissions are not uncommon. The semen is secreted by the testicles and then stored for future use in receptacles under the control of the spinal system of nerves. It is probable that the work of replenishing the evacuated contents of these receptacles goes on at once when needed, and not as some believe by constant secretion and as constant absorption, and in this way the supply and demand are kept balanced. The act of emission being a simple one of nervous explosion, brought about by nervous telegraphy, is performed by the titillation of the efferent nerves of the penis, which effect acts upon the spinal cord and as a result of this action, a direction to "fire" is flashed back over the afferent nerves, and the epileptic-like convulsion, called an ejaculation, is the result. Now when the means used to call forth this result are harsh, unnatural and frequently repeated, the finer nicety of adjustment is interfered with and the precision of action is destroyed or temporarily lost, and the result is that the "hair-trigger" action is destroyed. Then emissions occur on slight provocation. A full bladder, an undue amount of covering, slight erotic dreams, and unsound sleep, etc., will allow an emission. In the self-abuser the urine always passes in a ragged stream, which sometimes justifies the ambitious surgeon to cut out a stricture which exists, but not in the man who is cut.

The testicles and penis are flabby, and the former tender. Varicocele is the rule. After thirty-five the prostate is enlarged or tender, or both. The bowels, as a rule, are irregular or constipated. The contractile power behind the passing urine is lost, so that a few drops remain in the penis, and pass when a drop is least wanted. This is the state the masturbator finds himself in. He grows alarmed at the emissions, believing that if they could be stopped he would be well. As a rule, when they are stopped he remarks a scarcity of erections, and then his fears take on a new apparel, and he is full of forebodings.

Emissions, *per se*, amount to very little, unless excessive in times or amount. The causes which underlie them are harmful and call for attention. To stop the emission not only seems to be the desire of all patients, but most doctors direct their energy to this end, and so it happens that stomachs are filled with the bromides, lupulin, and many other drugs supposed to control this matter.

When advice is sought of the medical man, who looks upon such patients as licentious cowards and their condition a just reward for their ungoverned passion, the counsel takes the form of making light of the patient's fears, or a placebo is given, and the result is that in either case the patient is forced to the conclusion that the doctor does not understand the case, and then it is that the sleek gentry whose claims are paraded in the public print get in their work. This state of affairs is unfortunate. To say to masturbators that if they will stop the habit they will get well, is nonsense, and such opinion in any other line of practice would stamp the author as an ass. To say that violent and unnatural abuse of any system of organs is not most likely to occasion serious structural or functional derangement is to deny the very basis of our professional claim. When a set of organs having the close community of operation with mentality that the sexual organs have is at sixes and sevens, it is a misfortune that such medical men are in the path of health-seeking young men. The habit of masturbation is, nine times out of ten, contracted at an age when judgment is still dozing. To look upon these patients as deserving of the suffering they endure, or to make light of their distress, is alike narrow-minded. When advice is given, it should cover the points given by the editor in his answer to the query, and they all refer to improving the general health, to putting all the functions into the best possible working order. But there is one point that must never be forgotten, and that is the one that relates to the coördinating power between the organs of generation and the nervous control of the same. The organs directly concerned must be toned up, for it is a fact that the rest does not restore the lost tone and power, even if rest does stop the drain. It must never be forgotten that when a fairly developed varicocele exists, the restoration of testicular power will not come from rest alone.

Patients can do much by way of baths, exercise and the attention to the excretions that will build up the body, but in the newer materia medica such drugs as saw palmetto, phytolacca, black willow, viburnum, and others will do a wonderful work when rightly used in these cases. They will not alone restore the nervous break, but they will give relief to the mental inquietude which, after all, is the most melancholy part of the whole matter. The world of regret, mortification, and grief which ever and anon overwhelms the victim of his own lust, takes from life all the charm and from his future all the roseate dreams of home and happiness. To not only heal the victim's body but to also cure his mind, is a work both possible and desirable. And this I believe is capable of accomplishment. The possibilities in this field of therapeutics are great, but the rewards of faithful effort justify the belief that this large class can be, and surely ought to be, reclaimed from the irregulars who have reaped so richly and with so little return in substantial good.—JOHN A. DE ARMAND, M.D., Davenport, Iowa.—*Exchange.*

